# Clinical performance of a novel and rapid bioassay for detection of thyroid-stimulating immunoglobulins in Graves’ orbitopathy patients: a comparison with two commonly used immunoassays

**DOI:** 10.3389/fendo.2024.1469179

**Published:** 2024-09-27

**Authors:** Gijsbert J. Hötte, Maaike de Bie, Ronald O.B. de Keizer, P. Martijn Kolijn, Roosmarijn C. Drexhage, Sharon Veenbergen, Marjan A. Versnel, P. Martin van Hagen, Dion Paridaens, Willem A. Dik

**Affiliations:** ^1^ Department of Oculoplastic, Lacrimal & Orbital Surgery, Rotterdam Eye Hospital, Rotterdam, Netherlands; ^2^ Laboratory Medical Immunology, Department of Immunology, Erasmus MC University Medical Center Rotterdam, Rotterdam, Netherlands; ^3^ Department of Internal Medicine, section Endocrinology, Erasmus MC University Medical Center Rotterdam, Rotterdam, Netherlands; ^4^ Academic Center for Thyroid Diseases, Erasmus MC University Medical Center Rotterdam, Rotterdam, Netherlands; ^5^ Department of Immunology, Erasmus MC University Medical Center Rotterdam, Rotterdam, Netherlands; ^6^ Department of Internal Medicine, Section Allergy and Clinical Immunology, Erasmus MC University Medical Center Rotterdam, Rotterdam, Netherlands; ^7^ Department of Ophthalmology, Erasmus MC University Medical Center Rotterdam, Rotterdam, Netherlands

**Keywords:** Graves orbitopathy, disease activity, treatment response, methylprednisolone, TSI, functional bioassay

## Abstract

**Background:**

For the selective detection of thyroid-stimulating hormone receptor antibodies with stimulating properties (thyroid-stimulating immunoglobulins; TSI), a novel and rapid bioassay (Turbo TSI) has been introduced. We evaluate the clinical performance of Turbo TSI in Graves’ orbitopathy (GO) patients and compare it to a bridge-based TSI binding immunoassay and third generation TSH-R-binding inhibitory immunoglobulins (TBII) assay. Also, we investigate the association of Turbo TSI and TBII measurements with GO activity and severity, as well as response to intravenous methylprednisolone (IVMP), and compare results to previous findings on the bridge-based TSI binding immunoassay.

**Methods:**

Turbo TSI, TBII and bridge-based TSI binding immunoassay measurements were performed in biobank serum from 111 GO patients and control cases (healthy controls [HC; n=47], primary Sjögren’s disease [SD; n=10], systemic sclerosis [SSc; n= 10], systemic lupus erythematosus [SLE; n=10]). Clinical characteristics and response to treatment were retrospectively retrieved from GO patient files.

**Results:**

Turbo TSI had the highest sensitivity (97.3%) and negative predictive value (96.1%), while bridge-based TSI binding immunoassay showed the highest specificity (100%) and positive predictive value (100%). Differentiating GO patients from control cases, receiver operating characteristic (ROC) analysis showed an area under the curve (AUC) of 98.5%, 95.7% and 99.8% for Turbo TSI, TBII and bridge-based TSI binding immunoassay, respectively. Turbo TSI (p<0.001) and TBII (p<0.01) levels were higher in patients with active compared to inactive GO. Correlation with CAS was stronger for Turbo TSI (*r*=0.42) than TBII (*r*=0.25). No statistically significant differences were observed in IVMP responders vs. non-responders for Turbo TSI (p=0.092) and TBII (p=0.21). For identifying active GO, an AUC of 75% with Turbo TSI and 67% with TBII was found. For IVMP response, AUC was 66.3% with Turbo TSI and 62.1% with TBII. In multivariate logistic regression analyses, both assays were independently associated with disease activity (p<0.01 for both assays) and IVMP response (p<0.01 for Turbo TSI; p<0.05 for TBII).

**Conclusions:**

The new Turbo TSI functional bioassay has good clinical performance. Although turbo TSI is a stronger marker of activity and IVMP response than TBII, results are comparable to our previously published findings on the bridge-based TSI binding immunoassay.

## Introduction

Graves’ orbitopathy (GO) is an autoimmune inflammatory condition of the orbital soft tissues. GO is most often associated with hyperthyroidism in patients with Graves’ disease (GD) and is characterized by proptosis, eyelid retraction, edema, restricted ocular motility and diplopia, while 3-5% of cases experience loss of vision due to dysthyroid optic neuropathy or corneal breakdown (1). The pathogenesis of GO is still not fully understood, but involves orbital fibroblasts that express thyroid-stimulating hormone (TSH) receptor (TSH-R) and insulin-like growth factor 1 receptor (IGF-1R). Stimulatory autoantibodies against the TSH-R (TSH-R-Ab) cause activation of orbital fibroblasts, leading to inflammation, fibroblast proliferation, and production of glycosaminoglycans, which subsequently results in tissue expansion, edema and fibrosis (2, 3).

The clinical course of GO is characterized by an initial phase of active inflammation (active phase), which ultimately transitions into an inactive or burnt out phase (4). During the active phase, patients may benefit from immunosuppressive treatment, which aims to decrease inflammation and subsequently mitigate disease severity and residual symptoms (5). For this reason, identification of patients with active disease is important. However, the clinical scoring systems used to assess the degree of inflammation have certain limitations (5–7). Furthermore, approximately 40% of patients do not respond sufficiently to high dosage intravenous methylprednisolone (IVMP), which is still the first line immunosuppressive treatment, while they are exposed to the side effects. Consequently, these IVMP non-responders often require other immunosuppressive medication or radiotherapy to further stabilize the immune-inflammation (8).

To improve the assessment of GO activity and severity, and to identify IVMP non-responders prior to treatment, reliable biomarkers are required. Measurement of TSH-R-Ab is a sensitive tool to diagnose GD/GO (9–11) and, due to its central role in disease pathogenesis, also holds potential as a biomarker for monitoring GO progression and response to treatment. Based on their effect on TSH-R signaling two main categories of TSH-R-Ab are recognized: 1) TSH-R-stimulating antibodies (TSAb), also referred to as TSH-R stimulating immunoglobulins (TSI) and 2) TSH-R-blocking antibodies (TBAb), also referred to as TSH-R-blocking immunoglobulins (TBI) (12). TSH-R-Ab are commonly measured with a competitive-binding immunoassay where they compete with either TSH or a TSH-R monoclonal antibody for binding to TSH-R and are therefore referred to as TSH-R-binding inhibitory immunoglobulins [TBII, third generation]. However, these competitive-binding immunoassays provide no information on the biological activity of the TSH-R autoantibodies and typically measure the total of TSI and TBI (12). More recently, a bridge-based binding immunoassay was introduced that measures TSI more specifically, but not exclusively, and displays slightly better diagnostic performance in terms of sensitivity and specificity than competitive-binding immunoassays (10, 11, 13–17). In contrast, cell-based bioassays enable determination of functional activity of TSI or TBI, that can even co-exist or alternate during disease course in patients with GD (12, 18–21). Although binding immunoassays correlate with GO disease severity and activity, there are indications that cell based assays are more sensitive for detecting TSI than available binding immunoassays and correlate more closely with GO activity/severity (13, 16, 22–30). However, because of their technical complexity TSI bioassays are currently not used routinely in clinical practice. Only recently, a new commercially available TSI bioassay (Thyretain^®^ Turbo TSI Stimulating Reporter BioAssay) has been introduced, which significantly simplified and shortened the technical process compared to previously available TSI bioassays, making it more practical for routine use (results obtained within 2 hours) (31).

Therefore, the goal of our present study was to compare the clinical performance of the Turbo TSI bioassay with a third generation TBII and the bridge-based TSI binding immunoassay in patients with GO. Additionally, we investigate the association of the Turbo TSI bioassay and TBII measurements with GO disease activity, severity and response to intravenous methylprednisolone (IVMP) treatment, and compare this with results we previously obtained for the bridge-based TSI binding immunoassay (28).

## Methods and materials

### Patients and controls

For this study, serum samples that were stored at -80°C in the Combined Ophthalmic Research Rotterdam Biobank (CORRBI) were used. Ethical approval for CORRBI in general was granted by the local medical ethical committee (MEC-2012-031). Informed consent was obtained for all CORRBI participants after being informed on the ethical issues regarding storage and use of samples. The use of samples for our study was approved by the biobank committee. Files from patients whose samples were stored under the (tentative) diagnosis of GO were selected for further review. Clinical characteristics, laboratory tests and orbital imaging were evaluated to confirm diagnosis. In total, serum samples from 111 GO patients were included, as previously reported (28). Serum samples from a cohort of 47 healthy individuals were obtained as a control group, as approved by the local medical ethical committee (MEC-2021-0251). Additionally, we included three groups of patients with a confirmed diagnosis of non-thyroid autoimmune disease: primary Sjögren’s syndrome (SD; n = 10), systemic sclerosis (SSc; n = 10), systemic lupus erythematosus (SLE; n = 10) (MEC-2011-116 and MEC-2016-202).

### Clinical evaluation

Evaluation of patients and controls was performed as previously reported (28). Medical history and demographic features were recorded for all patients and controls. For GO patients, results from ophthalmological and orbital examination were retrospectively obtained from the patient files. Severity of the condition was determined using the EUGOGO classification (mild, moderate-to-severe, and sight-threatening GO) (5). Disease activity was assessed using the clinical activity score (CAS) of seven items: spontaneous retrobulbar pain, gaze evoked pain, eyelid erythema, conjunctival hyperemia, eyelid swelling, chemosis and inflammation of the caruncle/plica (6). Active disease was defined as a total CAS of ≥ 3 points in one or both eyes. Patients who were treated with IVMP after the biobank sample was obtained, were evaluated for treatment response. IVMP dosing schemes were based on EUGOGO guidelines and tailored in selected cases depending on comorbidity and side effects. For severe disease, the standard scheme included 1000mg of IVMP for three consecutive days, which was repeated if indicated. For moderate-to-severe disease, the standard dosing regimen consisted of a cumulative dose of 4500mg of IVMP in 12 weekly infusions. As part of a recent study by our group, a small subset of patients with moderate-to-severe disease was treated with a regimen of prednisolone-encapsulated liposomes (two times 150mg intravenously with a 2-week interval) (32). A beneficial response to IVMP treatment was defined as: 1) achievement of a total CAS < 3 in both eyes, or 2) an improvement of ≥ 2 points in one eye without concomitant deterioration in the fellow eye.

### TSH-R-Ab measurement

Serum samples were defrosted and analyzed under strict quality rules (ISO15189) by the Laboratory Medical Immunology at Erasmus MC. The automated bridge-based TSI binding immunoassay (Immulite^®^ 2000 TSI; Siemens Healthineers AG, Erlangen, Germany) was performed as previously reported in this cohort, with a cut-off of < 0.55 IU/L for negativity (28). For TBII, an automated competitive fluorescent enzyme-immunoassay EliA™ (Thermofisher Scientific, Freiburg, Germany) was used and < 2.9 IU/L was used as a cut-off for negativity. Turbo TSI bioassay kits were kindly provided by the manufacturer and performed according manufacturer’s instructions (Quidel, San Diego, Californica, USA). In short, reference, control and patient samples were added to a white 96-well plate in singlet. Turbo TSI cells were mixed with cAMP reagent and 50 μl of the cell suspension was added per well and incubated for 1 hour at room temperature. Thereafter luciferase signal was measured (GloMaX Explorer; Promega) and results were analyzed with the Turbo TSI-analysis tool and TSI concentration (IU/L) was calculated against the reference curve. A cut-off for negativity of < 0.0241 IU/L, as defined by the manufacturer, was used. For Turbo TSI bioassay, the highest concentration on the calibration curve was 11.293 IU/L. Samples with a concentration above this value were extrapolated using the manufacturers software. Samples that could not be extrapolated were defined as 1.5x the highest extrapolated concentration.

### Statistical analysis

Castor EDC was used as clinical data management system (33). Data were subsequently exported to SPSS v.28 (IBM corp., Armonk, New York, USA) and Prism (GraphPad Software, La Jolla, California, USA) for statistical analysis. Clinical sensitivity, specificity, positive predictive value (PPV) and negative predictive value (NPV) were calculated for all three assays, using the total of controls (i.e. healthy individuals and patients with non-thyroid autoimmune disease) as a reference. Sensitivity rates of the three assays were compared among the group of GO patients using McNemar test. Similarly, specificity rates were compared among the control subjects. Also, diagnostic odds ratio (DOR) was used as a single measure to define performance of the tests and was calculated as *(true positives/false negative)/(false positives/true negatives)* (34). Differences in continuous variables between groups were evaluated using Mann-Whitney U test. For categorical variables Fisher-Freeman-Halton exact or Pearson chi-squared test was used. Spearman rank correlation coefficient was used for correlation analyses. Receiver operator curve (ROC) analysis was performed and optimal cut-off values were calculated with Youden’s indices. Both univariate and multivariate logistic regression models were constructed.

## Results

### Patient characteristics

**Table 1** summarizes the demographic and clinical data of patients with GO (n = 111), as previously published (28), as well as the non-thyroid autoimmune disease groups (SD n = 10, SSc n = 10, SLE n = 10) and healthy controls (n = 47). Clinical data of GO patients correspond to the visit at which the biobank sample was obtained, including 14 (12.6%) with mild disease, 87 (78.4%) with moderate-severe disease and 10 (9.0%) with severe disease. In total 34 patients (30.6%) were smokers. Smoking status did not differ among severity groups (four smokers with mild GO, 26 with moderate-to-severe, and four with severe GO; p = 0.66). In total, 39 patients were treated with IVMP. After baseline, there was a further deterioration in one patient with moderate-to-severe GO, resulting in 11 patients ultimately being treated with methylprednisolone for severe disease (median cumulative dose 3000mg; IQR = 1000), while 24 patients received standard treatment for moderate-to-severe disease (median cumulative dose of 4500mg; IQR = 0) and another four patients with moderate-to-severe disease were treated with prednisolone-encapsulated liposomes (cumulative dose of 300mg) as part of a previously published study (32). Median time between obtaining the serum sample and the start of IVMP treatment was 11 days (IQR = 39.50). The median duration between completion of IVMP treatment and subsequent clinical evaluation was 16.5 days (IQR = 38.50).

**Table 1:** Demographic and clinical data of GO patients and control groups.

		Graves’ orbitopathy	Sjögren’s disease	Systemic Sclerosis	Systemic Lupus Erythematosus	Healthy controls	p-value
		(n = 111)	(n = 10)	(n = 10)	(n =10)	(n = 47)	
**Sex**	Male	37 (33.3%)	1 (10%)	2 (20%)	0 (0%)	11 (23.4%)	0.092
	Female	74 (66.7%)	9 (90%)	8 (80%)	10 (100%)	36 (76.6%)	
**Age (years)**	Median (IQR)	50 (IQR = 21)	59 (IQR = 11)	62 (IQR = 19)	50 (24.25)	42 (IQR = 24)	0.017
**Smoking status**	Smoker	34 (30.6%)	N/A	N/A	N/A	0 (0%)	<0.001
	Non-smoker	57 (51.4%)				47 (100%)	
	Unknown	20 (18.0%)				0 (0%)	
**Thyroid disease history**	Hyperthyroidism/Graves’ disease	103 (92.8%)					
	Hypothyroidism/Hashimoto	8 (7.2%)					
**Previous thyroid treatment**	Complete or partial thyroidectomy	11 (9.9%)					
	Radioactive iodine	25 (22.5%)					
**Current thyroid medication**	Block & replace	35 (31.5%)					
	Titration	16 (14.4%)					
	Thyroid hormone	37 (33.3%)					
	None	23 (20.7%)					
**Duration of symptoms (months)**	Median	10 (IQR 22)					
**CAS**	Median	2.0 (IQR 3)					
	Active (CAS ≥ 3)	45 (40.5%)					
	Inactive (CAS < 3)	65 (58.6%)					
	Unknown	1 (0.9%)					
**Severity**	Mild	14 (12.6%)					
	Moderate-severe	87 (78.4%)					
	Severe	10 (9.0%)					
**Response to IVMP**	Responder	22 (56.4%)					
	Non-responder	16 (41.0%)					
	Unknown	1 (2.6%)					

CAS,Clinical Activity Score; IVMP, intravenous methylprednisolone; IQR, interquartile range; N/A, not available.

a: Fisher-Freeman-Halton exact test.

b: Mann-Whitney U test.

c: Chi-square test.

### TSH-R-Ab detection and clinical performance for Turbo TSI, EliA TBII and bridge-based TSI binding immunoassay

**Figure 1:**
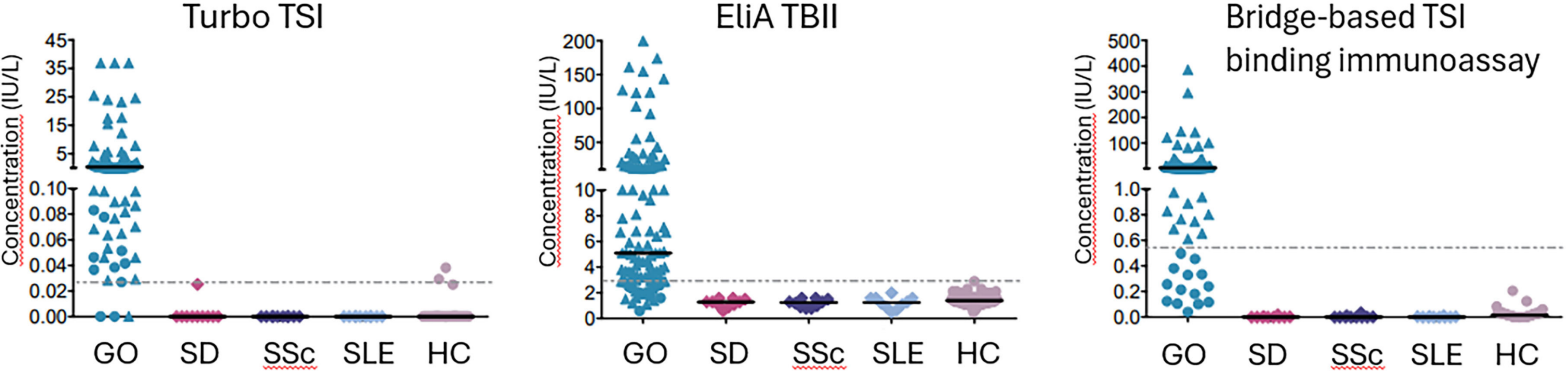
Distribution of antibody levels. Cut-off levels for negativity are shown as horizontal dashed lines: 0.0241 IU/L for Turbo TSI, 2.9 IU/L for TBII and 0.55 IU/L for bridge-based TSI binding immunoassay. Median concentrations are shown as solid line. Median concentrations and interquartile range, as well as percentages of positive/negative results, are also shown in **Table 2**. For Turbo TSI, the highest concentration on the calibration curve was 11.293 IU/L. Samples with a concentration above this value were extrapolated using the manufacturers software. Samples that could not be extrapolated were defined as 1.5x the highest extrapolated concentration. For the bridge-based TSI binding immunoassay, cases with a negative test result are depicted as dots, whereas positive results are depicted as triangles. The distribution of cases with a negative or positive result with the bridge-based TSI binding immunoassay are again shown as dots and triangles, respectively, in the graphs of TBII and Turbo TSI. Further details on the distribution of cases with a negative result in one test, but a positive result in the other tests, are shown in **Table 4**. GO, Graves’ orbitopathy; SD, Sjögren’s disease; SSc, Systemic sclerosis; SLE, Systemic lupus erythematosus; HC, healthy controls.

First, we investigated the analytical performance of all three assays. The distribution of antibody levels measured with the different assays in GO patients and controls are depicted in **Figure 1** and **Table 2**. In patients with GO, a strong correlation was observed between measurements with the three assays (**Table 3**). The highest negative result rate in GO patients was observed with EliA TBII (21.6%; **Table 2**). Of the TBII-negative GO patients, 95.8% and 62.5% tested positive with Turbo TSI and TSI bridge-based binding immunoassay, respectively (**Table 4**). With TSI bridge-based binding immunoassay, 14 negative results were obtained in GO patients (12.6%; **Table 2**), of which 85.7% tested positive with Turbo TSI and 35.7% with EliA TBII (**Table 4**). The lowest rate of TSH-R-Ab negative GO patients was obtained with Turbo TSI (2.7%; **Table 2**). Of these Turbo TSI-negative patients, one case also tested negative with EliA TBII and bridge-based TSI binding immunoassay, another had negative result with bridge-based TSI binding immunoassay but a slightly positive result with EliA TBII (3.0 IU/L), and the third case tested slightly positive with both immunoassays (1.07 IU/L with bridge-based TSI binding immunoassay and 3.5 IU/L with EliA TBII; **Table 4**).

**Table 2:** TSH-R-AB levels and negative/positive test results.

	Turbo TSI			EliA TBII			Bridge-based TSI binding immunoassay		
	Median IU/L (IQR)	Negative	Positive	Median IU/L (IQR)	Negative	Positive	Median IU/L (IQR)	Negative	Positive
Graves’ orbitopathy	0.38 (1.76)	3 (2.7%)	108 (97.3%)	5.1 (10.1)	24 (21.6%)	87 (78.4%)	3.36 (9.3)	14 (12.6%)	97 (87.4%)
Sjögren’s disease	0 (0)	9 (90%)	1 (10%)	1.3 (0.475)	10 (100%)	0 (100%)	0 (0.003)	10 (100%)	0 (100%)
Systemic sclerosis	0 (0)	10 (100%)	0 (100%)	1.25 (0.65)	10 (100%)	0 (100%)	0 (0.004)	10 (100%)	0 (100%)
Systemic lupus erythematosus	0 (0)	10 (100%)	0 (100%)	1.25 (0.75)	10 (100%)	0 (100%)	0 (0.004)	10 (100%)	0 (100%)
Healthy controls	0 (0)	44 (93.6%)	3 (6.4%)	1.4 (0.5)	46 (97.9%)	1 (2.1%)	0.016 (0.032)	47 (100%)	0 (100%)

Cut-off values were 0.0241 IU/L for Turbo TSI, 2.9 IU/L for TBII and 0.55 IU/L for Bridge-based TSI binding immunoassay.

IQR, interquartile range.

**Table 3:** Correlation between the different tests in patients with GO.

		Turbo TSI	EliA TBII	Bridge-based TSI binding immunoassay
Turbo TSI	Spearman coefficient		0.75	0.81
	p-value		<0.001	<0.001
EliA TBII	Spearman coefficient	0.75		0.88
	p-value	<0.001		<0.001
Bridge-based TSI	Spearman coefficient	0.81	0.88	
binding immunoassay	p-value	<0.001	<0.001	

**Table 4:** distribution of GO cases with a negative result in one test, but a positive result in the other tests.

	Turbo TSI positive	EliA TBII positive	Bridge-based TSI binding immunoassay positive
Turbo TSI negative (n = 3)		2 (66.7%)	1 (33.3%)
EliA TBII negative (n = 24)	23 (95.8%)		15 (62.5%)
Bridge-based TSI binding immunoassay negative (n = 14)	12 (85.7%)	5 (35.7%)	

When cut-off concentrations for positivity were applied as provided by the manufacturers, sensitivity was highest for Turbo TSI bioassay (97.3%), followed by the bridge-based immunoassay (87.4%) and EliA TBII (78.4%; **Table 5**). The higher sensitivity for both Turbo TSI bioassay and bridge-based TSI binding immunoassay compared to the TBII assay was statistically significant (McNemar test; both P < 0.001). Similarly, the difference in sensitivity was also statistically significant comparing Turbo TSI vs. Bridge-based TSI binding immunoassay (McNemar test; p = 0.003). Specificity was highest for the bridge-based TSI binding immunoassay (100%), followed by EliA TBII (98.7%) and Turbo TSI (94.8%; **Table 5**). Due to the absence of false positives with the bridge-based TSI binding immunoassay, statistical evaluation with McNemar test could not be performed to compare the specificity of the test with the other two assays. The difference in specificity of Turbo TSI bioassay and EliA TBII was not statistically significant (McNemar test; p = 0.38).

**Table 5:** Diagnostic accuracy of Turbo TSI, EliA TBII and bridge-based TSI binding immunoassay.

		Sensitivity	Specificity	PPV	NPV	DOR	AUC
Turbo TSI	cut-off 0.0241 IU/L^a^	97.3%	94.8%	96.4%	96.1%	657	98.5%
	cut-off 0.0261 IU/L^b^	97.3%	97.4%	98.2%	96.2%	1350	
EliA TBII	cut-off 2.9 IU/L^a^	78.4%	98.7%	98.9%	76%	275	95.7%
	cut-off 2.35 IU/L^b^	84.7%	98.7%	98.9%	81.7%	420	
Bridge-based TSI	cut-off 0.55 IU/L^a^	87.4%	100%	100%	84.6%	1042^c^	99.8%
binding immunoassay	cut-off 0.091 IU/L^b^	99.1%	97.4%	98.2%	98.7%	4325	

PPV, positive predictive value; NPV, negative predictive value; DOR, diagnostic odd ratio.

a = cut-off value provided by manufacturer.

b = optimal cut-off value based on Youden’s index.

c = true DOR could not be defined due to a false positive count of 0. An approximation of DOR was calculated by adding 0.5 to all counts.

Overall, the diagnostic performance of the Turbo TSI bioassay was higher than that of the EliA TBII assay, (DOR 657 vs. 275; **Table 5**). True DOR could not be defined for the bridge-based TSI binding immunoassay due to the absence of false positives with this assay. Therefore, an approximation of DOR was calculated by adding 0.5 to each cell of the contingency table (true positives, false positives, true negatives, false negatives), resulting in a DOR of 1042 (**Table 5**) (34).

For the differentiation of GO patients from the other cases, ROC analysis showed an area under the curve (AUC) of 98.5%, 95.7% and 99.8% for the Turbo TSI bioassay, EliA TBII and bridge-based TSI binding immunoassay, respectively (**Table 5**; **Supplementary Figure 1**). Calculated optimal cut-off concentrations, based on Youden’s index, were 0.0261 IU/L for the Turbo TSI bioassay, 2.35 IU/L for EliA TBII and 0.092 IU/L for the bridge-based TSI binding immunoassay (**Table 5**). With these cut-off values, overall diagnostic accuracy (i.e. DOR) was highest for the bridge-based TSI immunoassay (**Table 5**).

### Turbo TSI and EliA TBII measurements in relation to GO severity and activity

Previously we reported an association between the bridge-based TSI binding immunoassay and GO activity (but not severity) in the same cohort as described in this current study (28). Two other recent papers also demonstrated such an association with another FDA-cleared functional bioassay (Thyretain™) (7, 35). Therefore, we next investigated the association for Turbo TSI levels and TBII levels with disease severity and activity.

**Figure 2:**
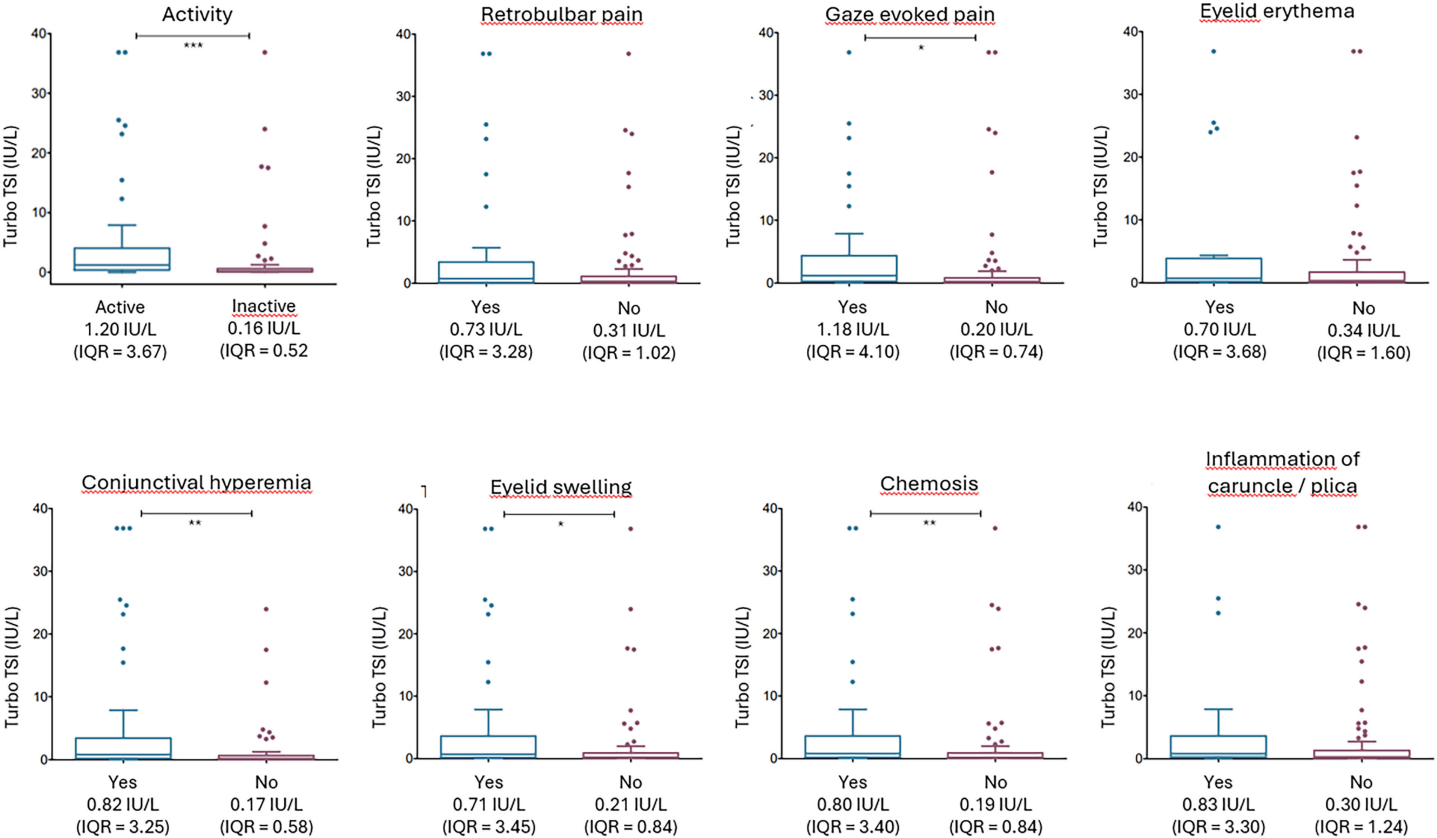
Turbo TSI measurements according to disease activity and the individual items of the CAS. TSH-R-Ab levels, measured with Turbo TSI, were significantly increased in patients with active disease (CAS ≥ 3) compared to inactive disease (CAS ≤ 2). Moreover, Turbo TSI measurements were significantly elevated in patients with gaze evoked pain, conjunctival hyperemia, eyelid swelling, chemosis and inflammation of caruncle/plica. * p < 0.05; ** p < 0.01; *** p < 0.001. Multiple comparisons correction was applied with Benjamini-Hochberg method.

**Figure 3:**
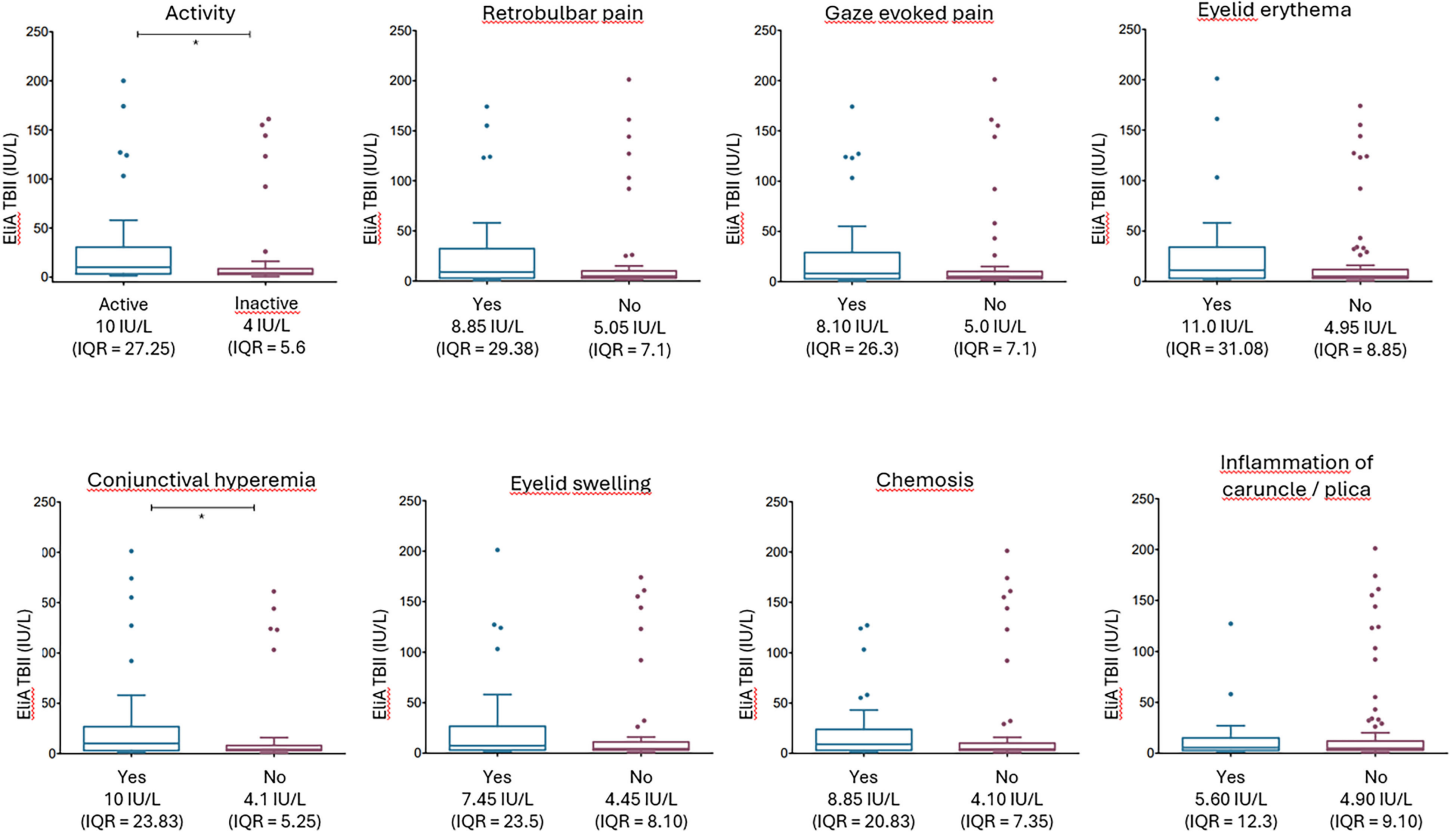
EliA TBII measurements according to disease activity and the individual items of the CAS. TSH-R-Ab levels, measured with EliA TBII, were significantly increased in patients with active disease (CAS ≥ 3) compared to inactive disease (CAS ≤ 2). Moreover, Turbo TSI measurements were significantly elevated in patients with conjunctival hyperemia, * p < 0.05. Multiple comparisons correction was applied with Benjamini-Hochberg method.

No differences in Turbo TSI levels and TBII levels were observed in relation to disease severity (**Supplementary Figure 2**). However, Turbo TSI levels and TBII levels were significantly higher in patients with active disease compared to patients with inactive disease (p < 0.001 and p < 0.01, respectively; **Figures 2**, **3**). Also, both assays correlated with total CAS, although the degree of correlation was stronger for the Turbo TSI bioassay (*r* = 0.42; p < 0.001) than for TBII (*r* = 0.25; p < 0.01). Moreover, the Turbo TSI bioassay correlated with several individual items of the CAS (gaze evoked pain [*r* = 0.30; p < 0.001], conjunctival hyperemia [*r* = 0.34; p < 0.001], eyelid swelling [r = 0.25; p < 0.01], chemosis [*r* = 0.31; p < 0.001] and inflammation of the caruncle/plica [*r* = 0.25; p < 0.01]), while EliA TBII only correlated with conjunctival hyperemia (*r* = 0.23; p < 0.05).

Because the individual CAS-items are dichotomous variables (yes/no), Spearman correlation may not sufficiently describe the association with TSH-R-Ab levels. Therefore, we additionally evaluated median TSH-R-Ab levels for each individual CAS-item (**Figures 2**, **3**). These findings corresponded well to the results from the Spearman correlation. After multiple comparison correction (Benjamini-Hochberg) Turbo TSI measurements were significantly elevated in patients with gaze evoked pain (p < 0.05), conjunctival hyperemia (p < 0.01), eyelid swelling (p < 0.05) and chemosis (p < 0.01; **Figure 2**), while EliA TBII measurements were only increased in patients with conjunctival hyperemia (p < 0.05; **Figure 3**).

**Figure 4:**
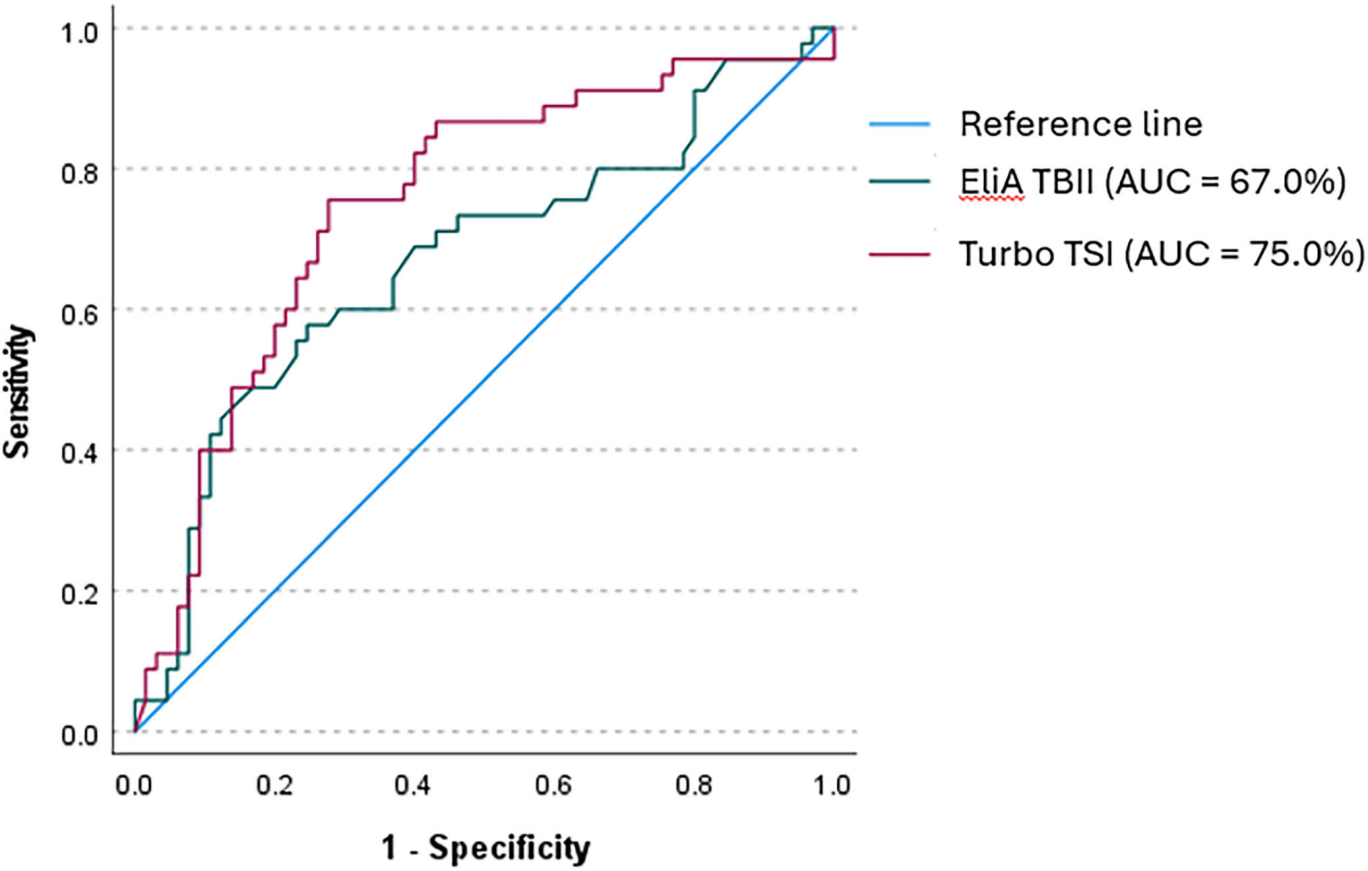
Receiver operator curve (ROC) for Turbo TSI and EliA TBII in identifying active disease. With Turbo TSI, ROC analysis showed an AUC of 75% for identifying patients with active disease. A cut-off value of 0.42 IU/L represented a 75.6% sensitivity and 72.3% specificity. With EliA TBII, the AUC was 67% and a cut-off value of 7.95 IU/L showed a sensitivity of 57.8% and a specificity of 75.4%.

ROC analysis for the Turbo TSI bioassay resulted in an AUC of 75% for identifying patients with active disease and a cut-off value of 0.42 IU/L, calculated with Youden’s index, was associated with 75.6% sensitivity and 72.3% specificity (**Figure 4**). For EliA TBII, the AUC for identifying active disease was 67% and a cut-off of 7.95 IU/L displayed a sensitivity of 57.8% and a specificity of 75.4% (**Figure 4**). These cut-off values were used for dichotomous distribution and subsequently applied to logistic regression models, showing that both high Turbo TSI and TBII measurements were associated with active disease, also when correcting for age, sex, smoking status, thyroid status and disease duration (**Table 6**). There was no significant collinearity among the variables used in this model (highest variance inflation factor [VIF] = 1.193).

**Table 6:** Univariate and multivariate logistic regression analyses for GO activity.

Turbo TSI		OR	CI (95%)	p-value
Univariate	**Turbo TSI (high)^a^**	**9.69**	**3.85 - 24.42**	**< 0.001**
Multivariate	**Turbo TSI (high)^a^**	**7.06**	**1.91 - 26.09**	**< 0.01**
	**Age**	**1.11**	**1.05 - 1.17**	**< 0.001**
	Sex (male)	0.60	0.14 - 2.45	0.47
	smoking (unknown)	0.21	0.028 - 1.63	0.14
	Smoking (yes)	1.43	0.34 - 6.00	0.62
	Hyperthyroidism	1.88	0.22 - 16.07	0.57
	Hypothyroidism	2.93	0.34 - 25.31	0.33
	Subclinical hyperthyroidism	2.01	0.46 - 8.72	0.35
	Subclinical hypothyroidism	2.04	0.21 - 20.22	0.54
	**Disease duration**	**0.90**	**0.84 - 0.97**	**< 0.01**
EliA TBII		OR	CI (95%)	p-value
Univariate	**EliA TBII (high)^b^**	**4.19**	**1.85 - 9.49**	**< 0.001**
Multivariate	**EliA TBII (high)^b^**	**6.55**	**1.59 - 26.91**	**< 0.01**
	**Age**	**1.11**	**1.05 - 1.16**	**< 0.001**
	Sex (male)	0.53	0.14 - 2.10	0.37
	smoking (unknown)	0.16	0.015 - 1.73	0.13
	Smoking (yes)	1.43	0.35 - 5.77	0.62
	Hyperthyroidism	1.67	0.20 - 13.63	0.63
	Hypothyroidism	2.77	0.30 - 25.75	0.37
	Subclinical hyperthyroidism	2.27	0.53 - 9.74	0.27
	Subclinical hypothyroidism	3.06	0.35 - 26.90	0.31
	**Disease duration**	**0.89**	**0.82 - 0.96**	**< 0.01**

a: High Turbo TSI is based on Youden’s index optimal cut-off for active disease (≥ 0.42 IU/mL).

b: High EliA TBII is based on Youden’s index optimal cut-off for active disease (≥ 7.95 IU/L).

Statistically significant variables are highlighted in bold.

### Turbo TSI and EliA TBII measurements in relation to response to intravenous methylprednisolone

Previously we reported a relation between TSH-R-Ab measured with the bridge-based TSI binding immunoassay and the response to IVMP (28). Therefore, we here also investigated the nature of this association for Turbo TSI bioassay and the EliA TBII immunoassay. No statistically significant difference was observed for the Turbo TSI when comparing IVMP non-responders (1.51 IU/L; IQR = 3.58) and responders (0.48 IU/L; IQR = 3.22, p = 0.092; **Supplementary Figure 3**). However, when the four patients who were treated with prednisolone-encapsulated liposomes were omitted from the analysis a statistically significant difference was observed (1.51 IU/L; IQR = 3.58 in non-responders vs. 0.36 IU/L; IQR = 1.57 in responders, p = 0.030). EliA TBII levels also did not differ between patients that did or did not respond to IVMP treatment (15 IU/L; IQR = 37.25 in non-responders vs 5.2 IU/L; IQR = 12.63 in responders; p = 0.21; **Supplementary Figure 3**), even when the analysis was performed without the four patients who were treated with prednisolone-encapsulated liposomes.

**Figure 5:**
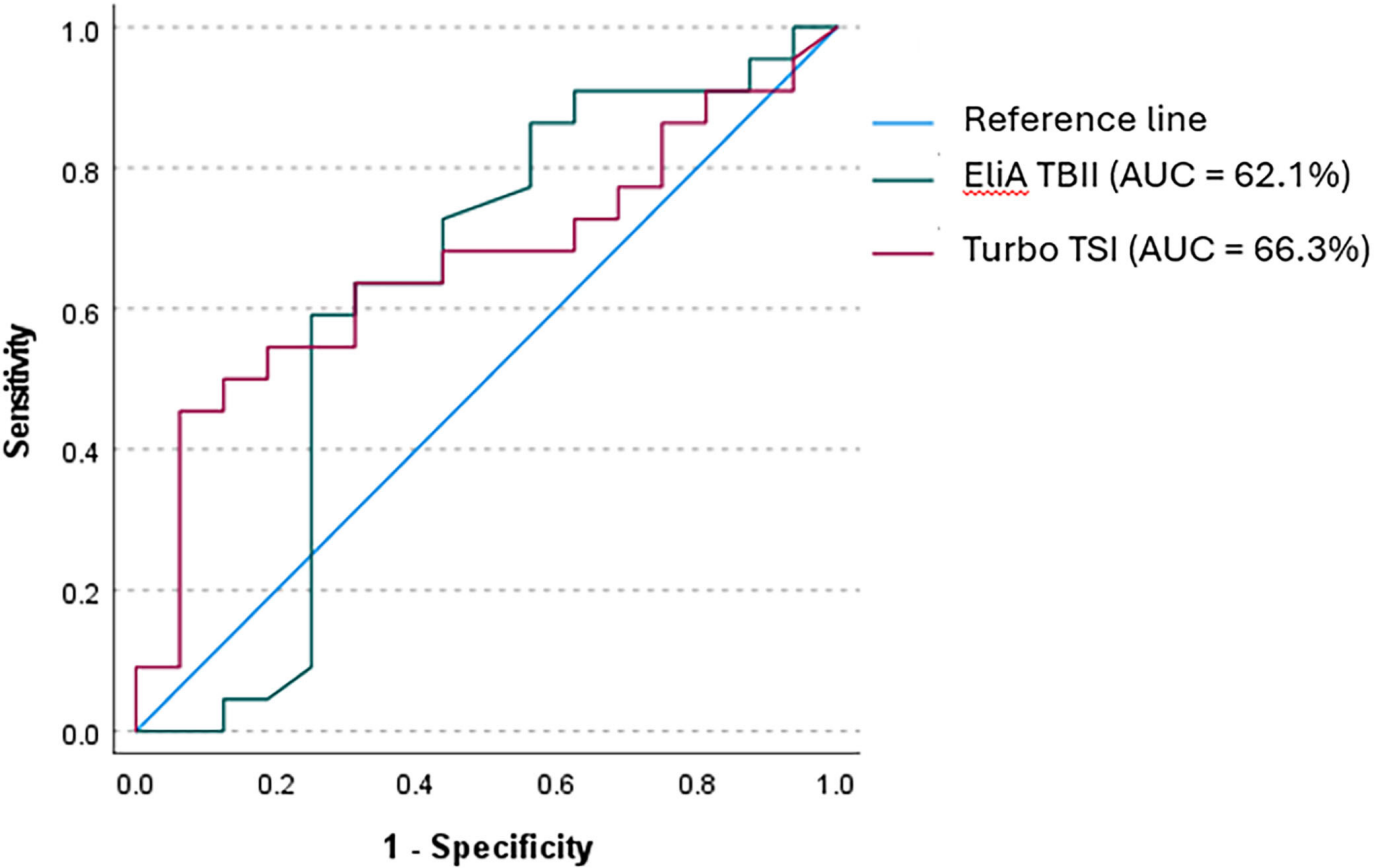
Receiver operator curve (ROC) for Turbo TSI and EliA TBII in identifying responders to IVMP. ROC analysis for identifying patients who would respond to treatment with IVMP showed an AUC of 66.3% for Turbo TSI, with 45.5% sensitivity and 93.7% specificity (cut-off 0.301 IU/L). For EliA TBII, an AUC of 62.1% was found, and a cut-off of 6.2 IU/L was associated with a sensitivity of 59.1% and specificity of 75%.

For Turbo TSI, ROC analysis showed an AUC of 66.3% for the identification of patients with a favorable response to IVMP. A cut-off value of 0.301 IU/L was associated with a 45.5% sensitivity and 93.7% specificity for predicting IVMP response (**Figure 5**). For TBII, the AUC for identifying patients with a favorable IVMP response was 62.1% and a cut-off of 6.2 IU/L represented a sensitivity of 59.1% and a specificity of 75% (**Figure 5**). Omission of the four patients who received prednisolone-encapsulated liposomes resulted in a similar AUC for TBII (63%), while the AUC for Turbo TSI increased up to 71.9%. The cut-off values were used for dichotomous distribution and subsequently applied to logistic regression models, showing that both high Turbo TSI and TBII measurements were associated with IVMP response, also when correcting for age, sex and smoking status (**Table 7**). There was no significant collinearity among these variables (highest VIF = 1.26).

**Table 7:** Univariate and multivariate logistic regression analysis for response to treatment with methylprednisolone.

Turbo TSI		OR	CI (95%)	p-value
Univariate	**Turbo TSI (low)^a^**	**12.5**	**1.40 - 111.84**	**< 0.05**
Multivariate	**Turbo TSI (low)^a^**	**61.06**	**2.92 - 1277.67**	**< 0.01**
	Age	0.96	0.90 - 1.028	0.27
	Sex (male)	0.14	0.013 - 1.54	0.11
	Smoking (unknown)	1.23	0.048 - 31.67	0.90
	Smoking (yes)	0.30	0.044 - 2.06	0.22
EliA TBII		OR	CI (95%)	p-value
Univariate	**EliA TBII (low)^b^**	**4.33**	**1.05 - 17.84**	**< 0.05**
Multivariate	**EliA TBII (low)^b^**	**12.87**	**1.30 - 127.05**	**< 0.05**
	Age	0.97	0.91 - 1.02	0.22
	Sex (male)	0.24	0.023 - 2.43	0.23
	Smoking (unknown)	3.29	0.19 - 55.64	0.41
	Smoking (yes)	0.39	0.068 - 2.26	0.30

a: low Turbo TSI is based on Youden’s index (< 0.301 IU/L).

b: low EliA TBII is based on Youden’s index (< 6.2 IU/L).

Statistically significant variables are highlighted in bold.

## Discussion

In this study we assessed the clinical performance of the newly developed Turbo TSI functional bioassay for TSI in patients with GO, and compared results with a third generation TBII immuno-binding assay (EliA TBII) and a bridge-based TSI binding immunoassay (Immulite TSI). Also, we investigated the association of Turbo TSI levels with disease activity, severity and response to IVMP in patients with GO.

All three assays performed well in differentiating GO patients from control cases (patients with SD, SSc, SLE and healthy controls) and a strong correlation was observed between results from the three assays. Using the cut-off values provided by the manufacturers, diagnostic performance based on DOR was higher with Turbo TSI compared to EliA TBII, which corresponds to reports comparing other TSI bioassays and TBII assays (15). For the bridge-based TSI binding immunoassay, true DOR could not be defined due absence of false positive cases. However, an approximation of DOR for the bridge-based TSI binding immunoassay revealed higher overall diagnostic performance compared to the other tests. More specifically, the bridge-based TSI binding immunoassay displayed higher sensitivity, specificity, PPV and NPV than EliA TBII, which corresponds to the findings from other studies (17, 36). In comparison to Turbo TSI, the bridge-based TSI binding immunoassay was associated with higher specificity and PPV, although sensitivity and NPV were lower. Moreover, ROC analysis for the differentiation of GO patients and control cases also showed highest AUC with bridge-based TSI binding immunoassay, although all three assays performed well.

These results suggest a slightly better overall diagnostic performance with the bridge-based TSI binding immunoassay compared to Turbo TSI. It must be noted, however, that the positive result that was observed with Turbo TSI in a patient with SD is not surprising since SD is associated with autoimmune thyroid disease, including Graves’ disease (37). Considering the fact that cell based bioassays are more sensitive for detecting TSI than binding immunoassays, we cannot exclude that this is a true TSI positive SD patient that was missed with the TSI bridge-based and TBII immunoassays (30). If that is the case, then incorrectly designating this patient as a false positive result for Turbo TSI leads to an underestimation of the specificity and PPV of this test, while incorrectly labeling this case as a true negative result for the bridge-based and TBII immunoassays leads to an overestimation of their specificity and NPV.

Interestingly, the optimal cut-off value for the bridge-based TSI binding immunoassay was found well below the level proposed by the manufacturer, which is in contrast to other studies that reported optimal cut-offs close to that of the manufacturer (10, 11, 38). A possible explanation is that, in contrast to our study, they used patients with other thyroid pathology as a control group, which may require higher serum TSH-R-Ab levels for accurate differentiation. Moreover, our study population consisted of patients with a relatively long disease duration, as well as a variety of treated and treatment-naïve cases, which may have resulted in lower overall TSH-R-Ab levels (4). However, in contrast to the bridge-based TSI binding immunoassay, the calculated optimal cut-offs for EliA TBII and Turbo TSI assays were close to the proposed cut-off concentrations. In that respect, it would also be interesting to further investigate the analytical performance and optimal cut-off concentration of Turbo TSI in a treatment-naïve cohort, as well as using other thyroid disease as a control group.

We recently reported that high TSI levels measured with the bridge-based TSI binding immunoassay are associated with active disease and IVMP treatment response, while no clear relation with disease severity was observed (28). In our current study we used the novel and rapid Turbo TSI bioassay, that measures TSI functional activity, to further explore these associations. Moreover, in order to investigate the reported superiority of bioassays over immunoassays as a biomarker in GO (22–26), we compared Turbo TSI results to a TBII assay, as well as to our previous findings with the bridge-based TSI binding immunoassay (28).

For both the Turbo TSI bioassay and the TBII assay we found an association with disease activity, but not with severity, both in univariate and multivariate analyses, confirming our observation made with the bridge-based TSI binding immunoassay. Yet, the Turbo TSI bioassay exhibited slightly better correlation with total CAS, and the individual CAS items, than the TBII assay. While we used single measurements, another study found a correlation between the decrease in Turbo TSI measurements and CAS improvement, which was also stronger than the correlation found for TBII, during experimental treatment with a neonatal Fc receptor targeting monoclonal antibody aimed at reducing the serum concentration of TSH-R-Ab (39). Also, we observed significantly higher Turbo TSI levels in patients with gaze evoked pain, conjunctival hyperemia, eyelid swelling and chemosis, while significantly higher TBII levels were only found in patients with conjunctival hyperemia. Furthermore, the discriminative value for identifying patients with active GO was higher for the Turbo TSI bioassay than TBII assay (AUC 75% vs. 67%). These results confirm previous reports demonstrating that TSI bioassays may serve as more effective biomarkers of disease activity than TBII assays (22–26). On the other hand, the correlation between Turbo TSI and CAS, as well as the discriminative performance of this assay in identifying patients with active GO, are more comparable to what we previously reported for the bridge-based TSI binding immunoassay (AUC 71%; an overview of the degree of correlation with CAS for all three assays is provided in **Supplementary Figure 4**), while Turbo TSI performed less well than what was previously reported with another FDA-approved functional TSI bioassay (Thyretain™; AUC 84.7%) in a patient cohort comparable to the cohort in our study (35).

Furthermore, univariate and multivariate logistic regression models showed that both Turbo TSI and TBII measurements were predictors of IVMP response, which is in line with our findings on the bridge-based TSI binding immunoassay (28). However, the confidence intervals were relatively wide, indicating low precision, which may be explained by the heterogeneity of our cohort. In contrast to our observation with the bridge-based TSI binding immunoassay, our current study showed no statistically significant difference in median Turbo TSI and TBII measurements between responders and non-responders to IVMP treatment, although increased Turbo TSI levels were observed in non-responders when four patients, who as part of a trial were treated with prednisolone-encapsulated liposomes, were omitted from the analysis. The discriminative performance of Turbo TSI and TBII in identifying IVMP responders is limited, corresponding to what we previously observed with the TSI bridge-based immunoassay. Of the three assays, bridge-based immunoassay (AUC 69% (28)) performed slightly better than Turbo TSI (AUC 66.3%), followed by TBII (AUC 62.1%). This illustrates the need to identify additional biomarkers for this clinically important application.

Previously, we proposed that the limited association between the bridge-based TSI binding immunoassay measurements and disease activity, as well as its limited ability to predict IVMP response, could be related to (co)detection of certain TBI, despite being marketed as TSI-specific (28). However, the results from our current study suggest that measurement of true TSI bioactivity with the Turbo TSI bioassay does not outperform the bridge-based TSI binding immunoassay as a biomarker for GO activity and for predicting IVMP treatment response. In that respect, it should however be noted that TSI bioassays measure the net TSH-R stimulatory activity, where blocking antibodies (TBI), when present, will still interfere with the assays measurement of (TSI) activity (16). This may also explain why two GO cases with a negative Turbo TSI test result tested positive with TBII and/or bridge-based TSI binding immunoassays. In these cases a simultaneous and more or less equal presence of both TBI and TSI may have resulted in neutral activity and hence a negative result with Turbo TSI bioassay, while the total of TBI and TSI was detected by TBII and bridge-based binding assays. Nevertheless, the net TSH-R stimulatory effect remains physiologically relevant because the thyroid gland, and orbital fibroblasts, are expected to respond to this net activity (3). However, the selective detection of stimulating, or blocking, autoantibody activity is perhaps not as pertinent in every clinical context as previously suggested (25). Given the pathophysiological mechanisms of GD and GO, it is highly likely that the majority of TSH-R-Ab measured with immunoassays in these patients have stimulating properties (3). According to this hypothesis, the enhanced clinical relevance of the Turbo TSI bioassay and the TSI bridge-based immunobinding assay could simply be attributed to a better sensitivity in comparison to TBII. Although it may come at the cost of increased false positives, the high sensitivity of the Turbo TSI may be especially beneficial in complex cases, or cases that present with GO in the context of euthyroidism and a negative TBII or TSI immunoassay test result.

Our study is limited by the heterogeneity of the study population, consisting of patients with GD-associated orbitopathy and Hashimoto thyroiditis (HT)-associated orbitopathy. TSI is not regarded a hallmark of HT. However, TSI are highly prevalent in patients with HT-associated orbitopathy (40) and our study thus reflects the clinical variety in which GO presents. Also, the study population included cases that had been treated for thyroid disease, as well as a limited number (n = 8) of treatment-naive cases. Thyroid regulation may influence antibody levels and may therefore have influenced the diagnostic performance of the assays (4). Additionally, antithyroid drugs can modulate immune response in a dose-dependent manner. However, while the patients receiving antithyroid drugs were known, information on the specific dosage of the medication given was often unavailable to us because endocrinological care and follow-up was often provided by other institutions. Other factors that may have influenced TSH-R-Ab levels or response to IVMP, such as the precise time of RAI and evaluation of hypercholesterolemia, were often unknown for the same reason (41). Also, the group of treatment-naïve cases consisted of an insufficient number of patients to be used adequately for subgroup analysis and there was a lack of a control group with non-autoimmune thyroid disease. To further compare the analytical performance of the different assays in future research, it would be interesting to include such a control group, as well as an analysis in a treatment-naïve cohort. Another point of consideration is the heterogeneity in IVMP dosing schemes. In support of this, the association between Turbo TSI measurements and IVMP response was more apparent when four patients, who as part of a trial were treated with prednisolone-encapsulated liposomes, were omitted from the analysis. Moreover, the different dosing schemes used in this study may have affected the clinical classification as IVMP responder or non-responder. In severe disease, the clinical evaluation after IVMP is necessarily closer to immunosuppressive treatment, leaving less time for the treatment to take effect, which may result in clinical classification as non-responder more frequently. On the other hand, the high dose used in these patients may cause clinical improvement more often than the lower dose used in moderate-to-severe patients. Another point of consideration is the relatively long disease duration, which may negatively affect treatment outcome as immunosuppressive treatment is most effective in an early stage of the disease (5). However, we did not observe a statistically significant difference in disease duration between IVMP responders (median 6 months; IQR = 9.25) and non-responders (median 4 months; IQR = 5; p = 0.44). Additional limitations include the statistically significant differences in age and smoking status between GO patients and controls. However, no difference in TSH-R-Ab levels were found according to smoking status with Turbo TSI (0.18 IU/L; IQR 1.35 in smokers vs. 0.48 IU/L; IQR 2.58 in non-smokers; p = 0.12) and EliA TBII (4.1 IU/L; IQR 8.50 in smokers vs. 5.15 IU/L; IQR 11.75 in non-smokers; p = 0.21), nor did age correlate with TSH-R-Ab levels (*r* = 0.081; p = 0.40 for Turbo TSI and *r* = 0.074; p = 0.44 for EliA TBII). Finally, the relatively small number of patients in the treatment response groups and in the mild and severe disease groups may have limited adequate statistical analysis. While severe disease is rather rare in general, the number of mild cases in our cohort is limited because the study reflects a tertiary referral center.

In conclusion, the newly developed Turbo TSI bioassay displays better clinical performance than the third generation EliA TBII assay used in this study. Although Turbo TSI also has higher sensitivity than the bridge-based TSI binding immunoassay, overall diagnostic performance was slightly better with the latter, while both assays have comparable performance as a biomarker for disease activity and for predicting IVMP treatment response in patients with GO.

## Data Availability

The datasets presented in this article are not readily available. Requests to access the datasets should be directed to g.hotte@oogziekenhuis.nl.

## Supplementary material

Supplementary Figure 1 ROC analysis for differentiating GO patients from control cases.

Supplementary Figure 2 Turbo TSI and EliA TBII measurements according to GO severity groups. For Turbo TSI, median levels were 0.163 IU/L in mild disease, 0.392 IU/L (IQR = 1.88) in moderate disease and 0.521 IU/L (IQR = 7.29) in patients with severe disease. For EliA TBII, median levels were 3.25 IU/L (IQR = 9.25) in patients with mild disease, 5.6 IU/L (IQR = 9.1) in moderate disease and 7.55 IU/L (IQR = 23.25) in severe disease. These differences were not statistically significant.

Supplementary Figure 3 TSH-R-Ab levels (measured with Turbo TSI and EliA TBII) in responders and non-responders to treatment with intravenous methylprednisoloneFor Turbo TSI, the concentration was 0.48 IU/L (IQR = 3.22) in responders vs. 1.51 IU/L (IQR = 3.58) in non-responders (p = 0.092). For TBII, median TSH-R-Ab levels were 5.2 IU/L (IQR = 12.63) in responders vs. 15 IU/L (IQR = 37.25) in non-responders (p = 0.21).

Supplementary Figure 4 Correlation matrix with CAS. Correlation with total CAS, as well as with individual items of the CAS, is shown for all three assays. Only statistically significant correlations are depicted. The correlation results with bridge-based TSI binding immunoassay are published by Hötte et al. [28].
