# Supplementary figures and images for: Clinical performance of a novel and rapid bioassay for detection of thyroid-stimulating immunoglobulins in Graves’ orbitopathy patients: a comparison with two commonly used immunoassays
Source: Front Endocrinol (Lausanne). 2024 Sep 27;15:1469179. doi: 10.3389/fendo.2024.1469179 (PMC11466803; doi:10.3389/fendo.2024.1469179)

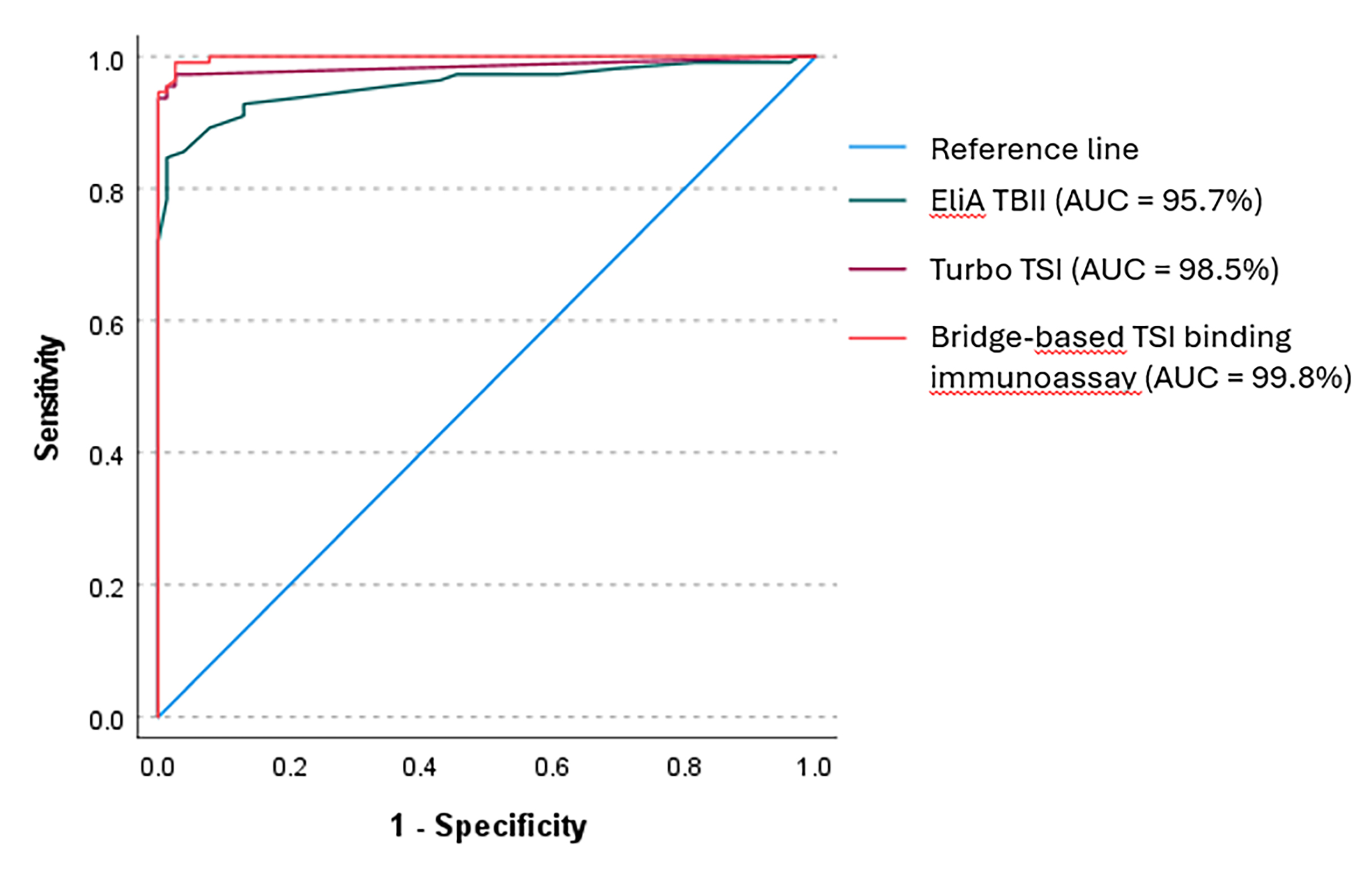

Supplement: Supplementary Figure 1 — ROC analysis for differentiating GO patients from control cases. [file Image1.jpeg]

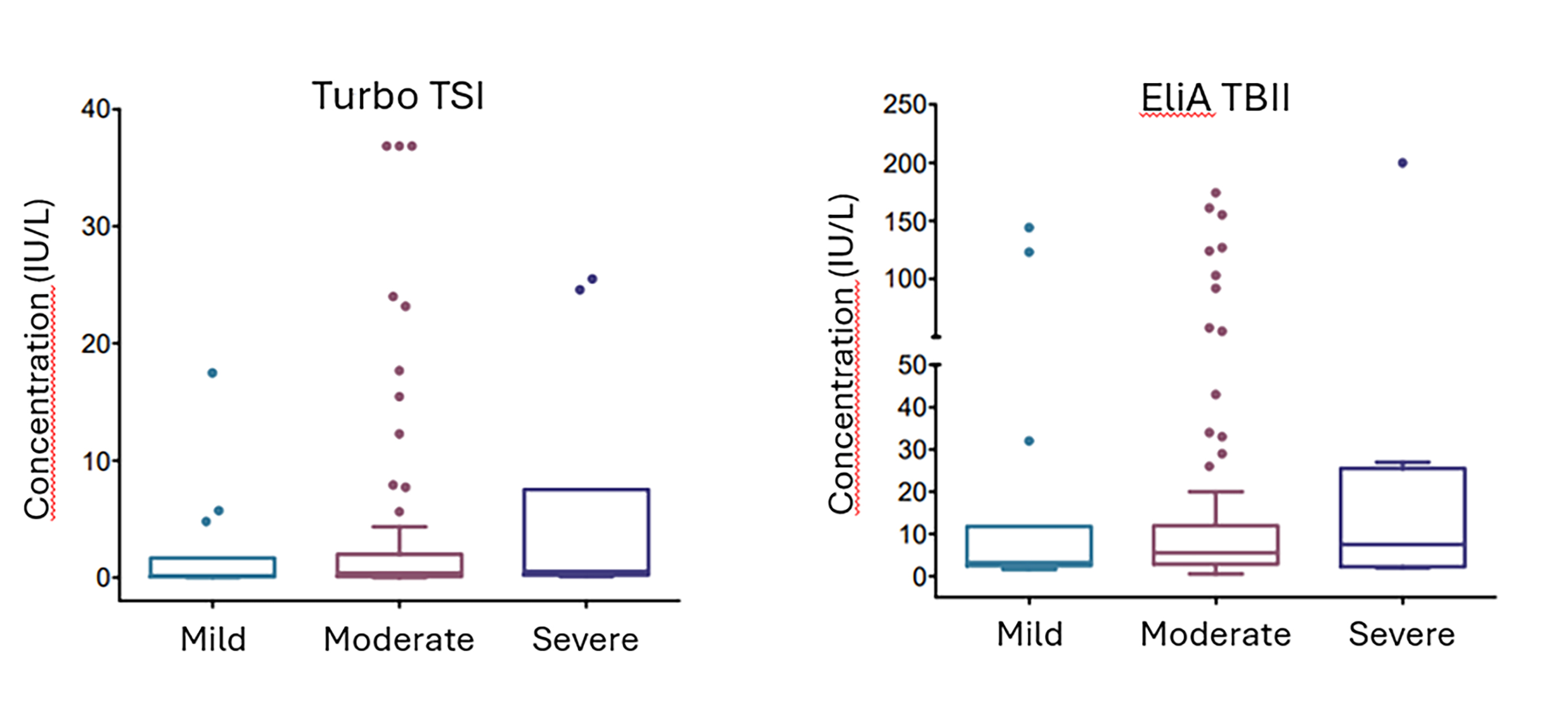

Supplement: Supplementary Figure 2 — Turbo TSI and EliA TBII measurements according to GO severity groups. For Turbo TSI, median levels were 0.163 IU/L in mild disease, 0.392 IU/L (IQR = 1.88) in moderate disease and 0.521 IU/L (IQR = 7.29) in patients with severe disease. For EliA TBII, median levels were 3.25 IU/L (IQR = 9.25) in patients with mild disease, 5.6 IU/L (IQR = 9.1) in moderate disease and 7.55 IU/L (IQR = 23.25) in severe disease. These differences were not statistically significant. [file Image2.jpeg]

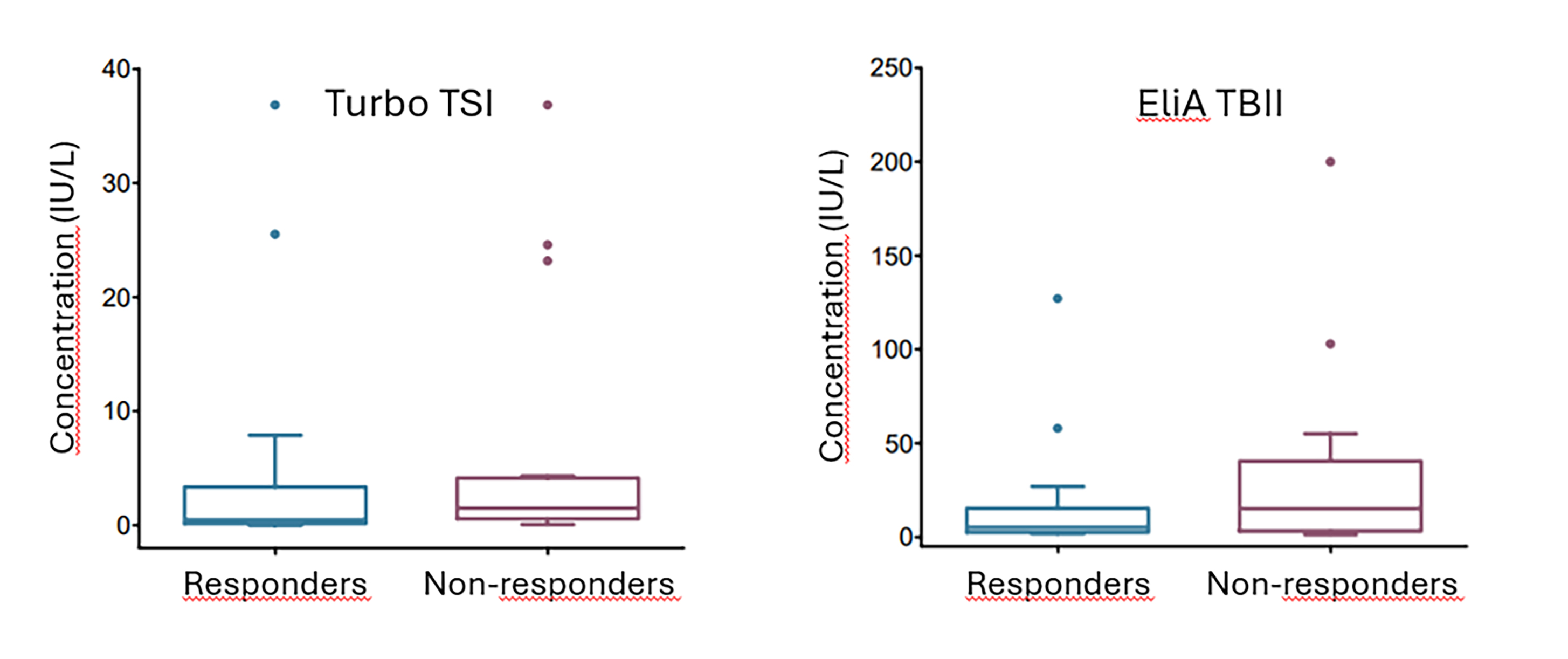

Supplement: Supplementary Figure 3 — TSH-R-Ab levels (measured with Turbo TSI and EliA TBII) in responders and non-responders to treatment with intravenous methylprednisoloneFor Turbo TSI, the concentration was 0.48 IU/L (IQR = 3.22) in responders vs. 1.51 IU/L (IQR = 3.58) in non-responders (p = 0.092). For TBII, median TSH-R-Ab levels were 5.2 IU/L (IQR = 12.63) in responders vs. 15 IU/L (IQR = 37.25) in non-responders (p = 0.21). [file Image3.jpeg]

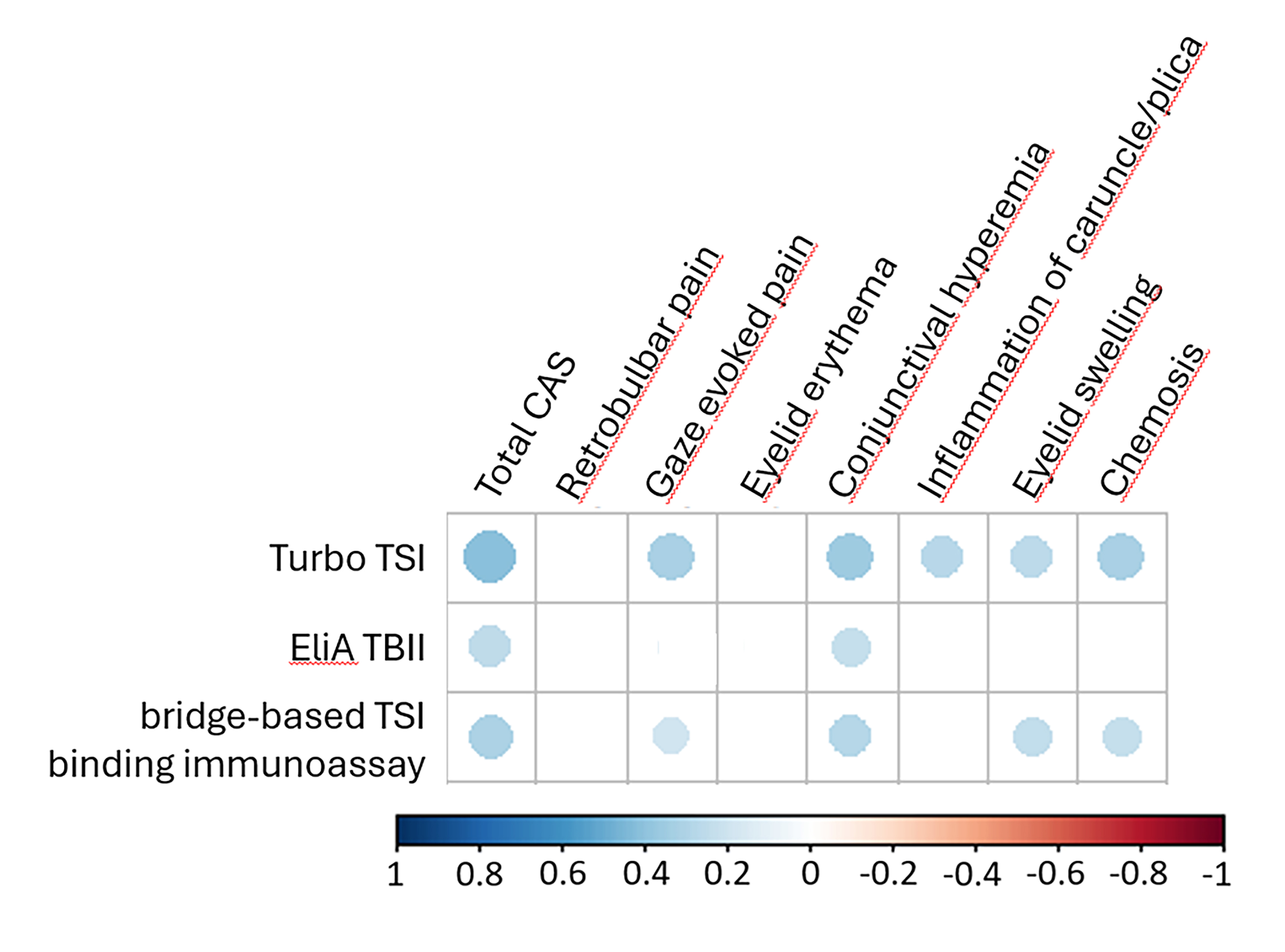

Supplement: Supplementary Figure 4 — Correlation matrix with CAS. Correlation with total CAS, as well as with individual items of the CAS, is shown for all three assays. Only statistically significant correlations are depicted. The correlation results with bridge-based TSI binding immunoassay are published by Hötte et al. [28]. [file Image4.jpeg]
